# Intensive Treatment of Lower-Limb Lymphedema and Variations in Volume Before and After: A Follow-Up

**DOI:** 10.7759/cureus.10756

**Published:** 2020-10-01

**Authors:** Jose Maria Pereira de Godoy, Maria de Fatima Guerreiro Godoy, Stelamarys Barufi, Henrique Jose Pereira de Godoy

**Affiliations:** 1 Cardiology and Cardiovascular Surgery, São José do Rio Preto School of Medicine (FAMERP), São José do Rio Preto, BRA; 2 Angiology and Vascular Surgery, Clínica Godoy, São José do Rio Preto, BRA; 3 Medicine, São José do Rio Preto School of Medicine (FAMERP), São José do Rio Preto, BRA; 4 Rehabilitation Service, Clínica Godoy, São José do Rio Preto, BRA; 5 General Practice, Clínica Godoy, São José do Rio Preto, BRA

**Keywords:** intensive treatment, lower-limb, lymphedema, godoy method, treatment, follow-up

## Abstract

Background and objective

Lymphedema is a clinical condition resulting from a failure in the drainage of the lymphatic system and the consequent formation of edema. The aim of the present study was to report the results of five days of intensive treatment for lymphedema and the maintenance of such results at the first follow-up evaluation.

Method

A clinical trial was conducted involving 409 patients with primary or secondary unilateral, lower-limb lymphedema who underwent intensive treatment with the Godoy method. The treatment consisted of eight hours per day of mechanical lymphatic drainage, 15 minutes per day of cervical stimulation, and compression mechanics involving the use of laced grosgrain (non-elastic) stockings alternated with bandages. Volumetric evaluations were performed at baseline, after five days of intensive treatment, and at the first follow-up evaluation performed one to three months after intensive treatment.

Results

The mean volume was 2,083.43 ml at baseline and 937.55 ml after five days of intensive treatment, corresponding to a 55% reduction. The mean was 800.83 ml at the follow-up evaluation, corresponding to a reduction of 137 ml [17.12 non-significant difference (p = 0.1)].

Conclusion

The intensive treatment of lymphedema leads to considerable reductions in edema within a short period of time, facilitating the continuity of treatment and demonstrating the credibility of the method.

## Introduction

Lymphedema refers to a clinical condition caused by a failure in the drainage of the lymphatic system and the resulting formation of edema. This condition may be primary or secondary. In primary cases, abnormalities in the lymphatic system are present at birth and may or may not lead to the development of lymphedema [1,2]. In secondary cases, the patient is born with an intact lymphatic system, but the damage caused to this system during the courses of life can lead to the development of lymphedema [3]. A recent concept divides secondary lymphedema into a hypertensive and non-hypertensive pattern, which is of fundamental importance to establish as to which drainage technique to use [4].

Complex physical therapy is the most widely used method for such cases [1-3]. However, novel concepts and treatment techniques have emerged in recent years, which propose the normalization or near normalization of lymphedema in all clinical stages, including elephantiasis [5-7]. Such concepts and methods enable the treatment of both upper-limb and lower-limb lymphedema.

The Godoy & Godoy method has undergone continual evolution, with the improvement of established concepts and the emergence of novel concepts. In 2019, the concepts of subclinical systemic lymphedema and systemic lymphedema associated with obesity and lipedema were described based on bioelectrical impedance analysis, contributing immensely to the understanding of the causes and treatment of lymphedema [8,9].

The intensive form of treatment represents a major advance in the therapeutic approach to these patients, enabling a 30-90% reduction in edema in five days for the majority of patients [7]. Therefore, the aim of the present study is to report the results of five days of intensive treatment for lymphedema and the maintenance of such results at the first follow-up evaluation.

## Materials and methods

Patients and setting

Charts of 409 patients with unilateral lower-limb lymphedema treated at the Godoy Clinic between 2014 and 2020 were evaluated.

Design

A clinical trial was conducted involving 409 patients with primary or secondary unilateral, lower-limb lymphedema who underwent intensive treatment with the Godoy method. Treatment consisted of eight hours per day of mechanical lymphatic drainage, 15 minutes per day of cervical stimulation, and compression mechanics involving the use of laced grosgrain (non-elastic) stockings alternated with bandages. Volumetric evaluations were performed at baseline, after five days of intensive treatment, and at the first follow-up evaluation performed one to three months after intensive treatment.

Inclusion criteria

Patients with unilateral lower-limb lymphedema in clinical stage II or III treated intensively for five days and then returned for evaluation after one to three months were included in the study.

Exclusion criteria

Patients with lower-limb lymphedema in clinical stage I, those with bilateral lymphedema, those with upper-limb lymphedema, and those from other countries were excluded from the study.

Randomization

Consecutive patients who met the eligibility criteria.

Statistical analysis

Data analysis involved descriptive statistics, the paired t-test, and the Mann-Whitney U test, considering an alpha error of 5%.

Ethical considerations

This study received approval from the Human Research Ethics Committee of the São José do Rio Preto School of Medicine (FAMERP), Brazil (#1.849.140).

Development

Charts of 409 patients with primary or secondary unilateral lower-limb lymphedema in clinical stages II or III who underwent intensive treatment using the Godoy method for five days were evaluated. The method consisted of eight hours per day of mechanical lymphatic therapy involving the RAGodoy® device, which performs passive movements of plantar flexion and extension, 15 minutes per day of cervical lymphatic therapy, which consists of gentle stimulation of the cervical region in a standardized manner, one hour per day of manual lymphatic therapy (Godoy Method) as well as the use of compression bandages and hand-crafted laced grosgrain stockings adjusted one to three times per day, as needed. Volumetric evaluations (water displacement) were performed at baseline, after five days of intensive treatment, and at the first follow-up evaluation performed one to three months after intensive treatment. The mean variations in the three volumetric evaluations were analyzed.

## Results

A total of 409 patients initiated treatment; five of them (1.22%) withdrew for personal reasons and 18 (4.4%) did not return for the follow-up evaluation. Sixty-one patients (14.9%) were men and 350 (85.5%) were women. The mean age of the patients was 43.2 years. One hundred twelve patients (27.3%) had secondary lymphedema and 72.6% had primary lymphedema.

**Figure 1:**
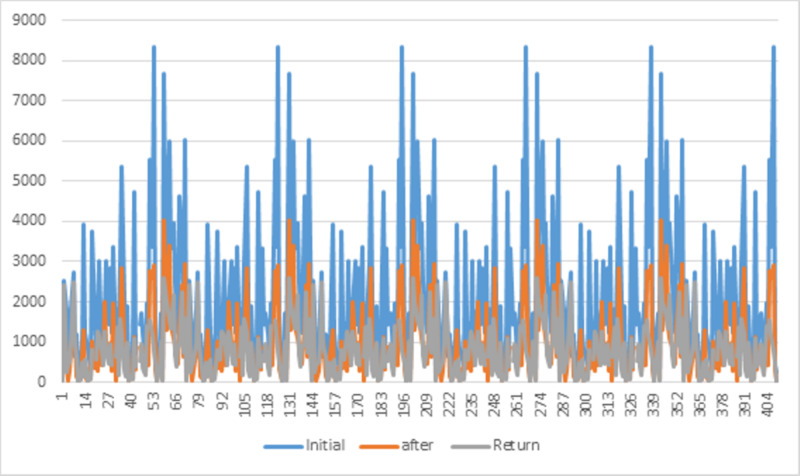
Variations in volume at baseline, after treatment, and at follow-up (patient’s return)

Table 1 displays the means at baseline, after five days of intensive treatment, and at the first follow-up evaluation. The mean volume was 2,083.43 ml at baseline and 937.55 ml after five days of intensive treatment, corresponding to a 55% reduction. The paired t-test revealed that this difference was statistically significant (p <0.0001). The mean was 800.83 ml at the follow-up evaluation, corresponding to a reduction of 137 ml (17.12%). However, the Mann-Whitney U test comparing mean volumes after five days of treatment and at follow-up evaluation revealed a non-significant difference (p = 0.1). Figure 1 illustrates the variations in volume before and after treatment as well as at the follow-up. Video 1 shows before and after treatment results.

**Video 1:** Godoy method

**Table 1:** Mean volume in ml at baseline, after five days of intensive treatment, and at follow-up (one to three months after intensive treatment)

Variables	Initial	After five days	Follow-up
Valid data	409	404	386
Mean	2,083.43	937.55	800.83
Standard deviation	1,774.73	889.07	655.61
Maximum	8,330	4,021	2,607
Median	1,515	733	628
Minimum	135	10	1

## Discussion

The present study demonstrated a significant reduction in the mean volume of the limb following intensive treatment for lymphedema in clinical stages II and III. Moreover, a further reduction in volume (17.12%) was found at the follow-up evaluation, although this reduction was non-significant. No other studies in the literature have reported this characteristic.

Intensive treatment is an important option for patients who live far away from the treatment center. This form of treatment provides a significant reduction that changes the lives of these patients in a short period of time. However, the Godoy Clinic adapts the treatment to the needs of each patient, which could be one to five eight-hour days per week, as performed in the present study.

All techniques employed were developed by the authors and scientific studies have confirmed their effectiveness in the treatment of lymphedema. The main form of treatment is with the RAGodoy® electromechanical device, which performs passive plantar flexion and extension for long periods. In the case of intensive treatment, the machine is used eight hours per day [10]. Cervical lymphatic therapy is a novel concept involving the stimulation of the lymphatic system that results in lymph drainage [5]. This stimulation consists of small movements on the skin in the cervical region, at the base of the neck, and in the supraclavicular region. Manual lymphatic drainage is performed in combination with mechanical therapy, generally after a substantial reduction in the volume of the limb. The laced, grosgrain (non-elastic) stocking is fundamental and is used six to eight hours per day [3,6,7]. 

The maintenance of the results is achieved by instructing the patients to continue to adhere to the different forms of treatment performed in the clinic. However, most patients only use the grosgrain stockings, whereas about 28% use this technique combined with mechanical lymphatic therapy. Adaptations are made based on the needs and possibilities of each patient. Phase 1 of treatment ends when normalization of the edema is achieved and is followed by phase II, which is the period of maintenance.

This study shows the impact of intensive treatment on the reduction in volume after the intensive period and at the first follow-up evaluation. The goal is a mean reduction of 70-80% in the edema, for which 10-15 days are needed. This is fundamental in cases of elephantiasis, for which such a reduction facilitates subsequent care. A goal for reaching normalization is set for each patient.

## Conclusions

Based on our findings, the intensive treatment of lymphedema leads to considerable reductions in edema within a short period of time, facilitating the continuity of treatment and demonstrating the credibility of the method.
